# Genders of patients and clinicians and their effect on shared decision making: a participant-level meta-analysis

**DOI:** 10.1186/1472-6947-14-81

**Published:** 2014-09-02

**Authors:** Kirk D Wyatt, Megan E Branda, Jonathan W Inselman, Henry H Ting, Erik P Hess, Victor M Montori, Annie LeBlanc

**Affiliations:** 1 Pediatric and Adolescent Medicine Residency Program, Department of Pediatrics, Mayo Clinic, 200 First St. SW, Rochester, MN 55905, USA; 2 Knowledge and Evaluation Research (KER) Unit, Mayo Clinic, 200 First St. SW, Rochester, MN 55905, USA; 3 Department of Health Sciences Research, Division of Health Care Policy and Research, Mayo Clinic, 200 First St. SW, Rochester, MN 55905, USA; 4 Division of Cardiovascular Diseases, Mayo Clinic, 200 First St. SW, Rochester, MN 55905, USA; 5 Division of Emergency Medicine, Mayo Clinic, 200 First St. SW, Rochester, MN 55905, USA; 6 Division of Endocrinology, Diabetes, Metabolism, Nutrition, Mayo Clinic, 200 First St. SW, Rochester, MN 55905, USA

**Keywords:** Gender, Shared decision making, Decision aids

## Abstract

**Background:**

Gender differences in communication styles between clinicians and patients have been postulated to impact patient care, but the extent to which the gender dyad structure impacts outcomes in shared decision making remains unclear.

**Methods:**

Participant-level meta-analysis of 775 clinical encounters within 7 randomized trials where decision aids, shared decision making tools, were used at the point of care. Outcomes analysed include decisional conflict scale scores, satisfaction with the clinical encounter, concordance between stated decision and action taken, and degree of patient engagement by the clinician using the OPTION scale. An estimated minimal important difference was used to determine if nonsignificant results could be explained by low power.

**Results:**

We did not find a statistically significant interaction between clinician/patient gender mix and arm for decisional conflict, satisfaction with the clinical encounter or patient engagement. A borderline significant interaction (p = 0.05) was observed for one outcome: concordance between stated decision and action taken, where encounters with female clinician/male patient showed increased concordance in the decision aid arm compared to control (8% more concordant encounters). All other gender dyads showed decreased concordance with decision aid use (6% fewer concordant encounters for same-gender, 16% fewer concordant encounters for male clinician/female patient).

**Conclusions:**

In this participant-level meta-analysis of 7 randomized trials, decision aids used at the point of care demonstrated comparable efficacy across gender dyads. Purported barriers to shared decision making based on gender were not detected when tested for a minimum detected difference.

**Trial registrations:**

ClinicalTrials.gov NCT00888537, NCT01077037, NCT01029288, NCT00388050, NCT00578981, NCT00949611, NCT00217061.

## Background

When shared decision making (SDM) is viewed as a conversation, clinician-patient communication styles lie at the crux. Clinicians and patients can act as barriers or facilitators to SDM, depending on the communication styles they choose to adopt [1]. It has been proposed that clinician and patient gender, and in particular the concordance or discordance between the gender of the clinician and patient, affects communication styles [2,3]. The limited evidence available on communication styles suggests that women may be more effective at behaviors associated with shared decision making. Their communication styles have been characterized as less dominant, with more awareness of others’ feelings [4]. Female physicians tend to engage more in partnership building, question asking, and information provision as opposed to male physicians; moreover, female patients tend to ask more questions, get more information, receive more counseling, engage in more emotional statements and are more involved in clinical encounters than male patients, although these findings have not been uniform across studies [2,5].

It seems reasonable to suggest that gender-associated communication style differences may impact SDM at the point of care. However, we know of no studies that systematically assess the impact of the clinician-patient gender grouping (“dyad”) on SDM during clinical encounters. The clinical trials of decision aids used at the point of care that we have conducted over the past 9 years were all performed in the same geographical area using similar study procedures and recorded many of the same outcomes. Because we had access to individual patient-level meta-analysis and this could be performed with minimal heterogeneity, we sought to measure the effect of clinician-patient gender dyads on the outcomes of decisional conflict, patient knowledge, provider engagement of the patient, patient satisfaction, and concordance between decision made and action taken among patients participating in randomized controlled trials where decision aids were used at the point of care using the single-step individual patient level meta-analysis.

## Methods

### Reporting

We will report the study according to the PRISMA Statement, where applicable [6]. We will also include additional criteria described by Riley et al. for reporting individual participant data meta-analysis [7]. The PRISMA Checklist, with the items recommended by Riley et al. appended, are included as Additional file 1.

### Study design

We conducted a participant-level meta-analysis to assess the extent to which clinician-patient gender groupings impact outcomes assessed within SDM-focused clinical trials where decision aids (DAs) were used during clinical encounters. A study protocol was not published, and the study was not registered in an online database. The Mayo Clinic Institutional Review Board approved each included study. For studies performed at Olmsted Medical Center (Decision Aids for Diabetes and Diabetes Choice Studies), approval was also obtained from the Olmsted Medical Center institutional review board. Each of the trials included in this study obtained written consent from patients and clinicians.

### Data source

We included all clinical encounters from all SDM trials conducted within the Knowledge and Evaluation Research Unit at Mayo Clinic in Rochester, MN, USA that were completed, but not necessarily published, at the time of the present study (Table 1) [8-12]. All of these trials took place between April 2005 and November 2010 were practice based, 2 arm, randomized controlled trials enrolling clinicians and patients at the point of care in Southeastern Minnesota, USA, practices, included a similar SDM intervention (i.e., a brief DA to be used by clinicians and patients during clinical encounters), assessed comparable measures of SDM processes and outcomes, and compared outcomes between an intervention (DA) and a control group. The AMI Choice study involved a nurse or nurse practitioner using the DA with the patient, while the remaining studies involved the DA being implemented by the patient’s physician. The rationale for not performing a systematic review to identify other includable studies was that we had access to individual encounter data and sufficient homogeneity of methods and outcomes to be combined, and addition of other studies would add heterogeneity.

**Table 1:** Study characteristics of the included trials

	**AMI Choice [under review]**	**Chest Pain Choice **[8]	**Decision Aids for Diabetes (DAD) **[9]	**Diabetes Choice **[10]	**Osteoporosis Choice I **[11]**/II [under review]**	**Statin Choice **[13]
**Trial registration**	NCT00888537	NCT01077037	NCT01029288	NCT00388050	NCT00578981	NCT00217061
					NCT00949611	
**Decision**	Intensive medical treatment to reduce 6-month mortality risk after acute myocardial infarction	Disposition after ruling out acute coronary syndrome for patients presenting with chest pain	Use of statins to reduce 10-year cardiovascular risk (Statin Choice) and choice of antihyper-glycemic medications (Diabetes Choice)	Choice of antihyper-glycemic medications	Use of bisphosphonate to reduce 10-year fracture risk among post-menopausal women	Use of statins to reduce 10-year cardiovascular risk (Statin Choice) in Patients with Type 2 Diabetes
**Setting**	Hospital	Emergency department	Primary care practices	Primary care practices	Primary care practices	Secondary care practices

The Acute Myocardial Infarction (AMI) Choice decision aid is a pamphlet that describes to hospitalized patients recovering from an acute myocardial infarction their personalized 6-month mortality risk without and with a bundle of medications that have been shown to improve mortality for the purpose of deciding whether the patient will take the medication bundle. The AMI Choice study [manuscript under consideration at a peer-reviewed journal], a patient level randomized trial, took place between March 2009 and June 2010 with follow up duration of 6 months. To be included in the study, patients had to be ages 18 to 90 years and recovering from an acute myocardial infarction. Patients were excluded if they could not give written informed consent or use the decision aid (including inability to read English, visual impairment, hearing impairment, dementia), were discharged to a nursing home, had do not resuscitate or comfort cares only order or died during the index hospitalization. Patients randomized to intervention were compared to usual care.

The Chest Pain Choice decision aid is a pamphlet that describes to patients presenting to the emergency with chest pain at low risk for acute coronary syndrome their personalized risk of having a heart attack or pre-heart attack within 45 days of their emergency room visit for the purpose of determining whether to be admitted to the emergency department observation unit for a cardiac stress test. The Chest Pain Choice study [8], a patient level randomized trial took place between February 2010 and November 2010 with a follow up period of 30 days. A multicenter trial is currently underway, but patients from the multicenter trial were not included in the meta-analysis. To be included in the study, patients had to be >17 years old presenting to the emergency department with symptoms on nontraumatic chest pain and be considered for admission to the emergency department observation unit for monitoring and cardiac stress testing within 24 hours. Patients were excluded if they had known coronary artery disease, elevated initial cardiac troponin T biomarker, had used cocaine within the previous 72 hours by history, or were pregnant. Patients randomized to intervention were compared to usual care.

The Diabetes Choice study [10] utilized the Diabetes Choice decision aid, which consisted of a series of “issue cards” that each lists an issue pertaining to the use of antihyperglycemic medications for type 2 diabetes mellitus (e.g., cost, side effects, daily routine) at the top and below compares the available medications according to the issue. Eligible patients were adults with a diagnosis of type 2 diabetes mellitus for at least one year who had a scheduled appointment with an enrolled clinician in the primary care setting and were able and willing to give informed consent to participate in the trial and were facing a decision about diabetes medications (as indicated by eligible glycated hemoglobin level conducted less than 6 months prior to enrollment measuring between 7.0% and 9.5% while taking 3 or fewer antihyperglycemic medications and not using insulin). The study, a clinician level randomized trial, took place between November 2006 and November 2007 with follow up of six months. Patients randomized to intervention were compared to usual care.

The Statin Choice decision aid lists the patient’s personalized 10-year risk of myocardial infarction with and without the use of a statin medication in addition to information about side effects associated with statin medications for the purpose of determining whether the patient will take a statin medication to reduce his or her cardiovascular risk. The Statin Choice trial [13], a clinician level randomized trial, took place in a subspecialty diabetes clinic between April and July 2005 with three month follow up. The trial included patients with a clinical diagnosis of type 2 diabetes who were able and willing to provide informed consent, had no reported contraindications to statin use and were available for three month follow up. After being randomized to receive the Statin Choice decision aid or a pamphlet on dyslipidemia, patients were further randomized to have their decision aid or pamphlet administered by their physician during the visit or by a researcher prior to the visit.

The Decision Aids for Diabetes (DAD) study [9] was a cluster randomized controlled trial randomized at the site level in primary care where patients were randomized to either the Diabetes Choice decision aid or Statin Choice decision aid and each group served as control for the other. The Diabetes Choice and Statin Choice decision aids used in the trial were essentially identical to the ones used in the Diabetes Choice and Statin Choice studies, respectively (above). The study took place between April 2010 and July 2011 with a six month follow up period. Eligible patients were ≥18 years of age, had a diagnosis of type 2 diabetes mellitus, recognized the participating clinician as their main diabetes care provider, had no major barriers to providing written informed consent, could communicate in English, were available for six-month follow up, and were facing the need to start or modify their current antihyperglycemic regimen.

The Osteoporosis Choice decision aid presents a postmenopausal woman’s individualized 10-year fracture risk with and without the use of bisphosphonate medications along with information about how the medications are taken and associated side effects and estimated cost for the purpose of determining whether the patient will take a bisphosphonate medication. The Osteoporosis Choice I trial [11], a patient level randomized trial, took place in a primary care setting between August 2007 and July 2008 with follow up of 6 months and included postmenopausal women aged 50 years or greater with bone mineral density levels consistent with osteopenia or osteoporosis who were not already taking bisphosphonates or other prescription medications for osteoporosis. To be included, patients must also be deemed eligible for bisphosphonate therapy by their clinician, have a follow up appointment with that clinician and be available for phone follow up at six months. Patients were excluded if they could not read English or had barriers to providing written informed consent or use of the decision aid. Patients who received the intervention were compared to usual care. The Osteoporosis Choice II trial, a patient level randomized trial, [manuscript under consideration at a peer-reviewed journal] took place in a primary care setting between May 2009 and April 2010 with follow up of 6 months. It was a three-arm study comparing the Osteoporosis Choice decision aid with provision of the patient’s World Health Organization Fracture Risk Assessment Tool (FRAX) score and usual care. Inclusion and exclusion criteria were identical to the Osteoporosis I trial [11].

### Data extracted

All enrolled participant data from each clinical trial was extracted for analysis from the primary study databases, which we had direct access to. Measures, including demographic information, were collected from post-encounter and follow-up surveys from patients and clinicians, pharmacy records, medical record review, and third-party video observation of the clinical encounters.

### Outcome measures

Patients’ level of decisional conflict was assessed immediately after consultations using the Decisional Conflict Scale (DCS) in paper form [14]. The scale includes 5 subscales and 16 items on a 0–4 Likert scale, where scores can be reported globally or for each subscale individually. We reported the scores for each subscale, transposing them to a 0–100 range with higher scores indicating greater comfort with decision making. The number of subscales assessed varied across trials. Only the subscales deemed by investigators to be most pertinent to the decision at hand were used in the original studies to prevent questionnaires from placing excess study burden on participants. The AMI Choice trial assessed 2 of the 5 subscales (Information and Effectiveness), the DAD trial assessed 3 of the 5 (Information, Effectiveness and Support), and the remaining trials assessed all 5 subscales.

Knowledge was assessed through patient self-report after the clinical encounter. Patients answered true/false knowledge questions pertaining to information considered essential in the decision-making context for the clinical problem at hand, mainly around cognizance of the problem, its alternatives, and associated benefits and risks. AMI Choice did not assess knowledge in this context and therefore is not included in this analysis. The total knowledge scores were expressed as a percent of the maximum possible score.

Patient engagement was measured using the OPTION scale, a third-party observer scale which evaluates clinicians’ efforts to involve patients in decision making [15]. Scores were assessed by a single reviewer after calibration of assessments with other reviewers on a smaller set of encounters. The scale has 12 items scored on a 0–4 scale which are then added to form the total score (maximum = 48). We transposed this score to a 0–100 range, with higher scores indicating greater involvement in decision making, for ease of interpretation. This is reported only in cases where recording of the encounter was conducted; reasons for non-recording included patient decline, clinician decline, equipment malfunction/unavailability, and encounter not conducive to recording.

Satisfaction with the encounter was measured immediately after the encounter using patients’ willingness to recommend the way they made the decision (i.e., use of the DA) to others from a questionnaire, measured on a 7-point Likert scale, converted into two categories: recommend (1–2) or not (3–7).

Chart reviews and pharmaceutical records provided evidence about the action patients took (i.e., actual decision) as extracted by a single study coordinator, which we compared to their declared decision on post-visit questionnaires (i.e., stated decision). Concordance is coded as complete agreement versus not, where complete indicates that the patient’s action was completely consistent with the decision made (e.g., start new medication was the decision, and a new medication was found within pharmacy records).

All data was carefully examined for inconsistencies at the time of the primary analysis, including verification of recorded survey results and consistency across reporting mechanisms.

### Statistical analysis

All analyses were conducted according to the intent to treat principle with encounters analyzed as randomized. Meta-analysis was performed using the one-step approach with individual encounter data; no pooled data were used in the analysis [7,16]. Analysis was conducted for all trials as patient randomized, ignoring clustering in the three trials. This was due to the low level of clustering effect found in the trials (DAD Intra Cluster Correlation, ICC = 0 for all outcomes, Diabetes ICC = 0 to 0.01 for all outcomes except OPTION ICC = 0.3, and Statin Choice ICC = 0.04 to 0.09 for all outcomes except OPTION ICC = 0.2. Categorical data are presented as counts and frequencies and continuous outcomes as means and standard deviations. Difference in baseline characteristics between gender groupings were analyzed with Wilcoxon-Mann–Whitney tests or Fisher’s exact tests as appropriate. Education level was missing at random for 4% of the population; values were imputed and utilized for analysis. The remainder of the dataset was complete.

We used the Higgins I^2^ coefficient to quantify the proportion of inconsistency in interventional effects across trials not explained by chance (i.e., reflecting true differences in trial results) alone [17]. Tests were conducted on each outcome to determine if the effect of the intervention (DA) arm versus the usual care (UC) arm differed between the six trials. The values for the coefficient for each outcome were as follows: DCS informed = 58.5%, DCS values = 48.8%, DCS support = 23.5%, DCS certainty = 66.8%, DCS effectiveness = 45.7%, Knowledge = 0.0%, Satisfaction = 44.0%, OPTION = 88.9% and Concordance = 42.7%. Random effects models were therefore performed to account for the heterogeneity found, thus results provided for an average treatment effect. The two-level mixed model was adjusted by fixed effects of age, education and type of clinician, interaction of gender mix and arm and a random effect of study.

Each continuous outcome was examined and found to have a normal distribution and was modeled as such. Collinearity was not found in the variables included in the model, verified by variance inflation factor. Binary outcome models were examined for goodness of fit using the Hosmer-Lemeshow method and there was no evidence of a poor fit. The continuous outcome models were found to be overall significant with a low R^2^ (<0.3), which indicates that the adjusted variables are not a good predictor of the outcome. As we were modeling to test the theory that gender mix has an impact on the outcomes and we used the factors that was available for the data, no further action was taken on this. The results are representative of the interaction of the intervention arm (DA versus UC) by the gender groupings being conducted or not. Results of the meta-analysis were reported using the adjusted mean differences (DA-UC) with 95% CI for each continuous outcome (DCS and subscales, general knowledge, and adherence). For binary outcomes the adjusted predicted percentage difference (DA-UC) with 95% CI were reported. Each model is adjusted by not only the study arm, gender grouping (male clinician/male patient, female clinician/female patient, same gender [i.e., combination of former two groups], male clinician/female patient and female clinician/male patient) and the interaction of them but also by patient age (continuous), education level (high school or less, more than high school) and clinician type (staff physician, resident/fellow physician, mid-level provider). Coefficients used in the model, as well as estimates for intercepts, residual variance on the consultation level and study level, are included as Additional file 2. The interaction between intervention and gender groupings were tested using the -2 log likelihood with a two-sided hypothesis test and significance reported at p-values of < 0.05. A sensitivity analysis was conducted as part of the pre-specified analysis plan to see if clinician gender was significantly related to the outcomes of interest. A random effects model was conducted using the same adjustment factors as above except substituting gender mix with clinician gender. Clinician gender was not found to be significantly related to any of the outcomes (results not shown). The gender mix was explored at 4 levels (male clinician/male patient, female clinician/female patient, male clinician/female patient, female clinician/male patient) to ensure that the same gender mixes (male clinician/male patient, female clinician/female patient) did not differ from each other. Therefore, it was decided to report gender mix at 3 levels (male clinician/female patient, female clinician/male patient and same gender clinician and patient).

Because the most prevailing source of bias was study selection, included studies were not individually assessed for risk of bias. Given the small number of studies, meaningful funnel plots could not be generated to assess between-study risk of bias.

We estimated the minimal important difference (MID) following as recommended by Sloan and Norman by considering the MID as equivalent to half of a standard deviation for each measure [18]. This value provided guidance on interaction effects and whether we could determine true non difference or the study was underpowered for the outcome.

To determine the extent to which a clinician participating multiple times impacted our results, we conducted a sensitivity analysis by subsetting the population to the first encounter for each clinician and the last encounter for each clinician. We modeled both subsets using the same techniques as above and found no impact on results. Results for the subset sensitivity analysis are not shown for this reason. All analyses were conducted using SAS 9.2 and STATA 12.1. Study data were recorded and managed using the Research Electronic Data Capture (REDCap) system [19].

## Results

A total of 775 encounters took place in the 6 trials conducted between 2005 and 2012. A higher percentage of encounters with the gender mix of female clinician/male patient were in the DA arm (71%). The distribution of gender mixes was imbalanced due to the AMI Choice trial in which only female clinicians took part in the DA arm. Moreover, nurses and mid level providers, most of them from the AMI Choice trial, contributed to a higher female clinician/male patient ratio than in other gender mix groupings (40% vs. range 2-15%, Table 2). The Osteoporosis Choice trial only enrolled female patients. Over 95% of patients enrolled in each study were white.

**Table 2:** Participant socio-demographic characteristics

	**Female clinician: Male patient (N = 109)**	**Male clinician: Female patient (N = 284)**	**Same gender (N = 382)**	**p value**
**Study**				<.0001
AMI Choice	40 (36.7%)	18 (6.3%)	48 (12.6%)	
Chest Pain Choice	22 (20.2%)	91 (32.0%)	91 (23.8%)	
DAD	11 (10.1%)	22 (7.7%)	70 (18.3%)	
Diabetes Choice	11 (10.1%)	29 (10.2%)	45 (11.8%)	
Osteoporosis Choice I	0 (0.0%)	49 (17.3%)	51 (13.4%)	
Osteoporosis Choice II	0 (0.0%)	47 (16.5%)	32 (8.4%)	
Statin Choice	25 (22.9%)	28 (9.9%)	45 (11.8%)	
**Arm**				<.0001
Control	32 (29.4%)	156 (54.9%)	196 (51.3%)	
DA	77 (70.6%)	128 (45.1%)	186 (48.7%)	
**Age: Mean (SD)**	60.3 (12.0)	62.4 (11.6)	60.6 (11.3)	0.15
**Patient education**^**1**^				0.18
HS or Less	41 (37.6%)	85 (29.9%)	133 (34.8%)	
Some college/Voc.	30 (27.5%)	112 (39.4%)	141 (36.9%)	
4 year/Graduate Degree	38 (34.9%)	87 (30.6%)	108 (28.3%)	
**Type of clinician**^**2**^				<.0001
Staff physician	37 (33.9%)	203 (71.5%)	246 (64.4%)	
Resident/fellow physician	28 (25.7%)	74 (26.1%)	79 (20.7%)	
Mid-level provider or nurse	44 (40.4%)	7 (2.5%)	57 (14.9%)	

Abbreviations: *DAD* Decision Aids for Diabetes, *SD* standard deviation, *HS* high school, *VOC* vocational.

^1^the values of 40 cases that were previously missing were imputed using single imputation.

^2^not unique clinicians, number based on patient encounters.

Because the same data as that used in the completed study was used for the individual participant data meta-analysis, our results were comparable to the final results for each individual study. No additional patients were added and no patients were removed for this analysis.

Clinicians could participate in each trial multiple times and could participate in multiple trials; the range of encounters where a clinician participated was 1 to 28. One patient participated in three studies (Chest Pain Choice, Osteoporosis Choice I, and Diabetes Choice), and two patients participated in two trials (Chest Pain Choice and Diabetes Choice; Decision Aids for Diabetes and Diabetes Choice). Sixty clinical encounters took place at a different medical center than the rest (50 encounters from the Decision Aids for Diabetes study and 10 from the Diabetes Choice study), and sufficient patient identifying information was not available to determine if any of these patients participated in any other included trials.

### Patient decisional conflict

**Figure 1:**
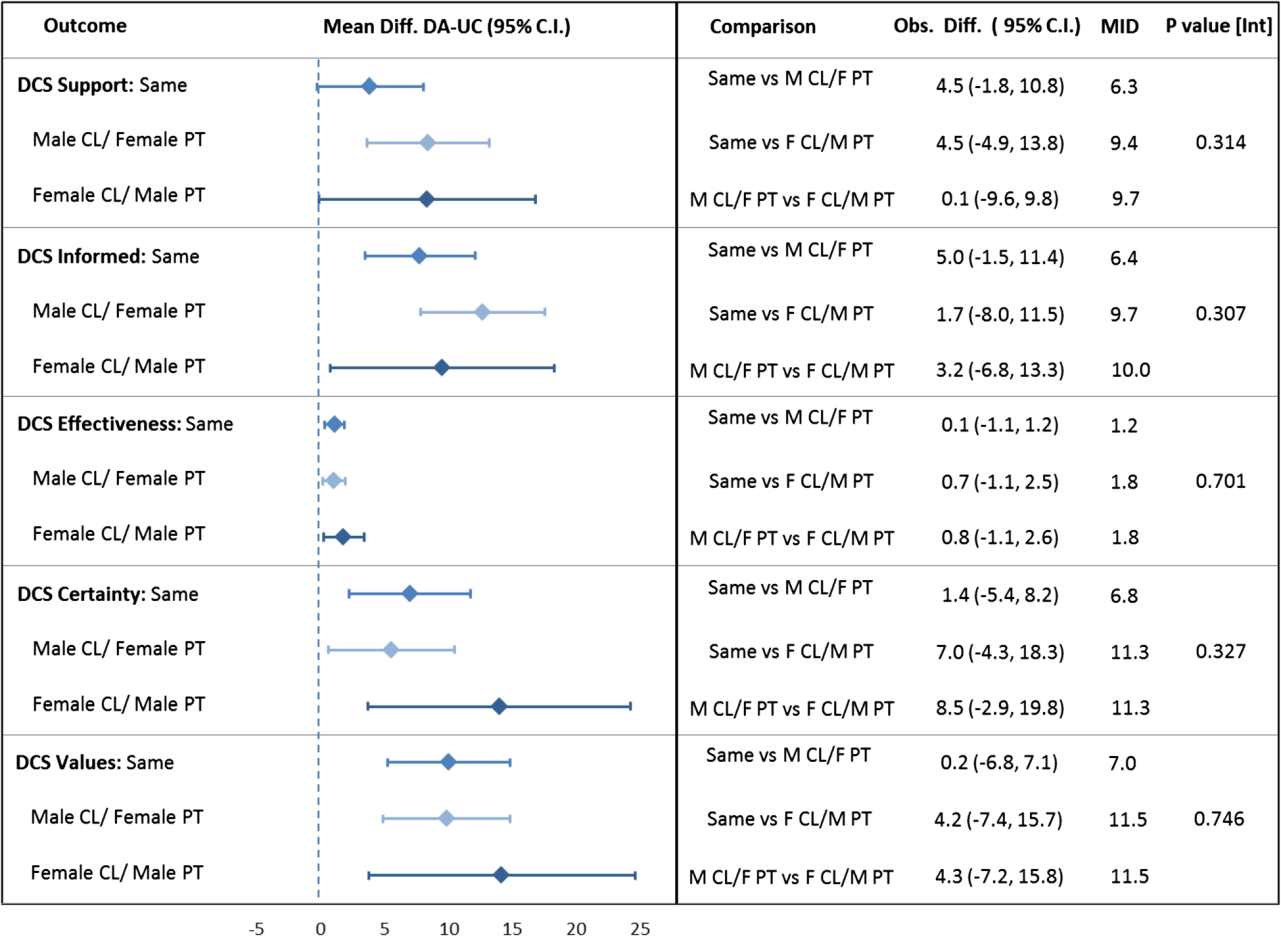
**Effect of gender groupings on decisional conflict.** Analysis separating the “same” gender group into male/male and female/female (not shown) made no change in the results. Footnote: Abbreviations: CL, clinician; PT, patient; MID, minimal important difference. Mean differences (DA – UC) calculated using simulation estimates from multilevel mixed-effects linear regression models for each arm within each gender mix. Mean differences were compared between gender mixes to test against MID.

While the DA had a marked improvement over UC in the informed, effectiveness, certainty and values subscales (Table 3) in the DCS across all gender mixes, no effect was seen in the support subscale, aside from in the male clinician/female patient gender mix (Figure 1). Interaction between gender mix and arm was not found to be significant among any of the subscales. The MID for each subscale was not met.

**Table 3:** Mean differences in outcomes between arms according to gender mix

**Outcome**	**Gender Mix**	**N (N (DA))**	**Mean Diff**^**a **^**(DA – UC) (95% CI)**	**Adj. Mean Diff**^**b **^**(DA - UC) (95% CI)**
DCS Support	Same	344 (172)	3.8 (-0.1, 7.7)	3.9 (-0.3, 8.0)
	Male CL/Female PT	258 (121)	8.3 (3.6, 12.9)	8.4 (3.6, 13.2)
	Female CL/Male PT	104 (74)	8.4 (-2.0, 18.9)	8.3 (-0.2, 16.8)
DCS Informed	Same	347 (172)	8.3 (4.2, 12.5)	7.8 (3.4, 12.1)
	Male CL/Female PT	259 (122)	12.4 (7.5, 17.3)	12.7 (7.8, 17.6)
	Female CL/Male PT	104 (74)	9.3 (-0.1, 18.7)	9.5 (0.7, 18.3)
DCS Effective	Same	346 (172)	0.8 (0.1, 1.6)	1.1 (0.3, 1.8)
	Male CL/Female PT	259 (121)	0.9 (0.0, 1.8)	1.0 (0.1, 1.9)
	Female CL/Male PT	104 (74)	1.8 (0.1, 3.4)	1.8 (0.2, 3.3)
DCS Certain	Same	251 (126)	6.8 (2.1, 11.5)	7.0 (2.2, 11.7)
	Male CL/Female PT	233 (115)	5.5 (0.3, 10.6)	5.5 (0.6, 10.4)
	Female CL/Male PT	56 (34)	12.6 (3.1, 22.1)	14.0 (3.7, 24.3)
DCS Values	Same	251 (126)	10.9 (5.9, 15.9)	10.0 (5.2, 14.8)
	Male CL/Female PT	233 (115)	9.8 (4.7, 15.0)	9.9 (4.9, 14.8)
	Female CL/Male PT	56 (34)	12.3 (1.4, 23.1)	14.2 (3.7, 24.6)
Knowledge	Same	291 (153)	13.3% (7.7, 18.9)	13.7% (7.7, 18.6)
	Male CL/Female PT	248 (120)	15.4% (9.4, 21.5)	15.4 (9.5, 21.2)
	Female CL/Male PT	62 (38)	20.3% (8.6, 32.1)	21.0% (9.1, 32.9)
Engagement (OPTION)	Same	231 (120)	19.5 (14.6, 24.5)	19.4 (15.9, 22.8)
	Male CL/Female PT	205 (99)	17.4 (12.1, 22.8)	19.0 (15.3, 22.6)
	Female CL/Male PT	56 (33)	16.8 (8.6, 25.0)	19.1 (12.2, 26.1)
Satisfaction	Same	340 (170)	15.9 (6.2, 25.6)	18.4 (7.4, 29.5)
	Male CL/Female PT	254 (122)	8.0 (-3.2, 19.2)	8.1 (-4.2, 20.3)
	Female CL/Male PT	101 (70)	17.7 (-1.8, 37.1)	19.8 (-1.4, 41.0)
Concordance	Same	287 (144)	-8.9 (-19.3, 1.6)	-6.2 (-15.1, 2.7)
	Male CL/Female PT	202 (103)	-17.7 (-30.1, -5.2)	-16.2 (-28.7, -3.7)
	Female CL/Male PT	94 (66)	10.0 (-10.4, 30.3)	7.6 (-9.2, 24.5)

Abbreviations: *DCS* decisional conflict scale, *M* male, *F* Female, *CL* clinician CL, *PT* patient.

^a^Unadjusted comparison.

^b^Adjusted by fixed effects of age, education and type of clinician, interaction of gender mix and arm and a random effect of study.

### Knowledge

**Figure 2:**
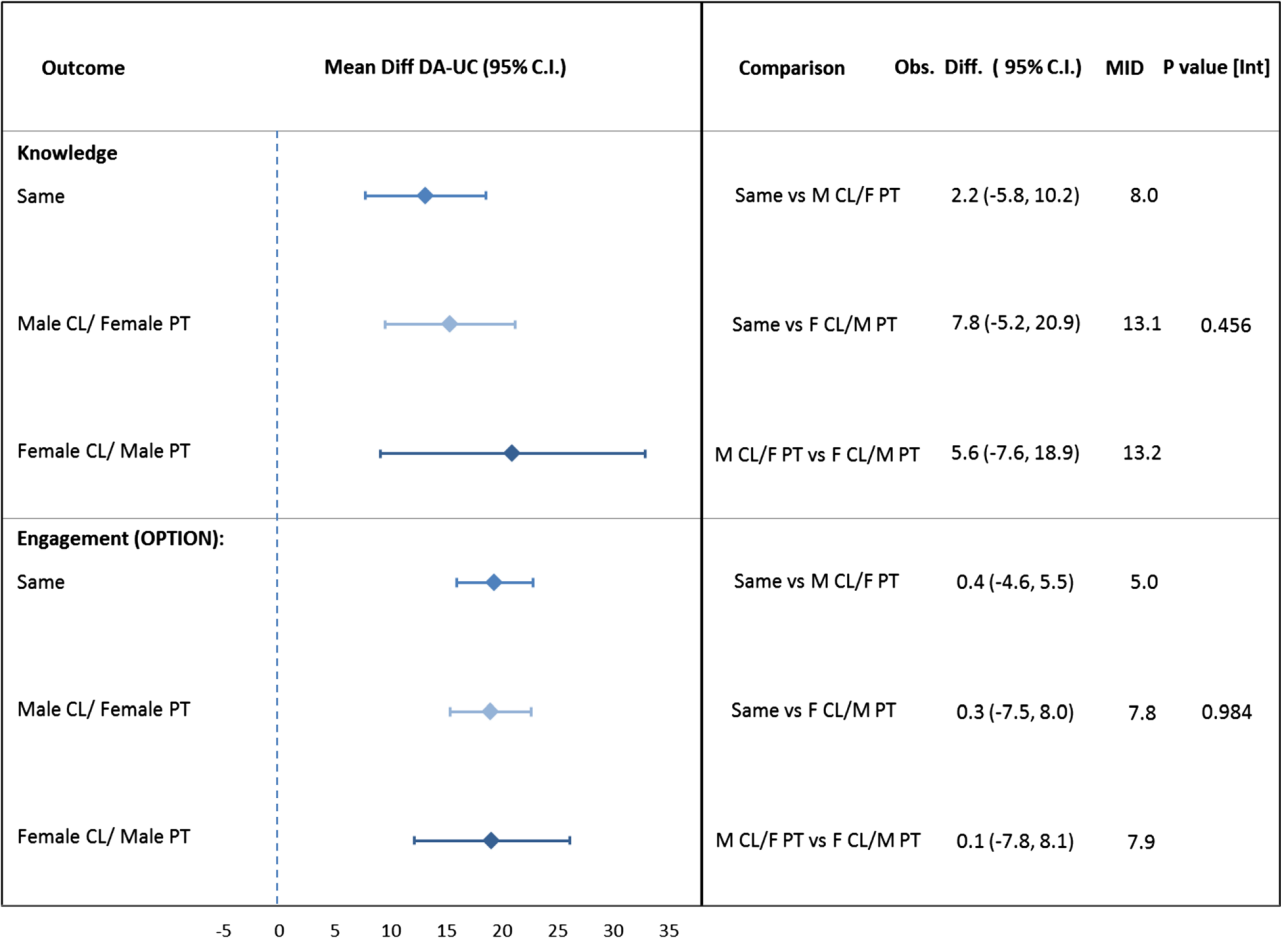
**Effect of gender groupings on knowledge and patient engagement (OPTION).** Analysis separating the “same” gender group into male/male and female/female (not shown) made no change in the results. Footnote: Abbreviations: CL, clinician; PT, patient; MID, minimal important difference. Mean differences (DA – UC) calculated using simulation estimates from multilevel mixed-effects linear regression models for each arm within each gender mix. Mean differences were compared between gender mixes to test against MID.

Knowledge scores were significantly higher for patients within the DA arm over usual care (Table 3) within each of the gender mixes. No effect of interaction between the intervention and gender mix was seen (Figure 2). The MID for knowledge was not met.

### Clinician engagement of patients

A significant increase in the extent clinicians engaged patients in the decision making process (i.e., OPTION score) for the DA arm was seen among all gender mixes (Figure 2). The impact of the intervention across the gender mixes was consistent, and no significant between gender mix differences were seen. The MID for clinician engagement of patients was not met.

### Satisfaction

**Figure 3:**
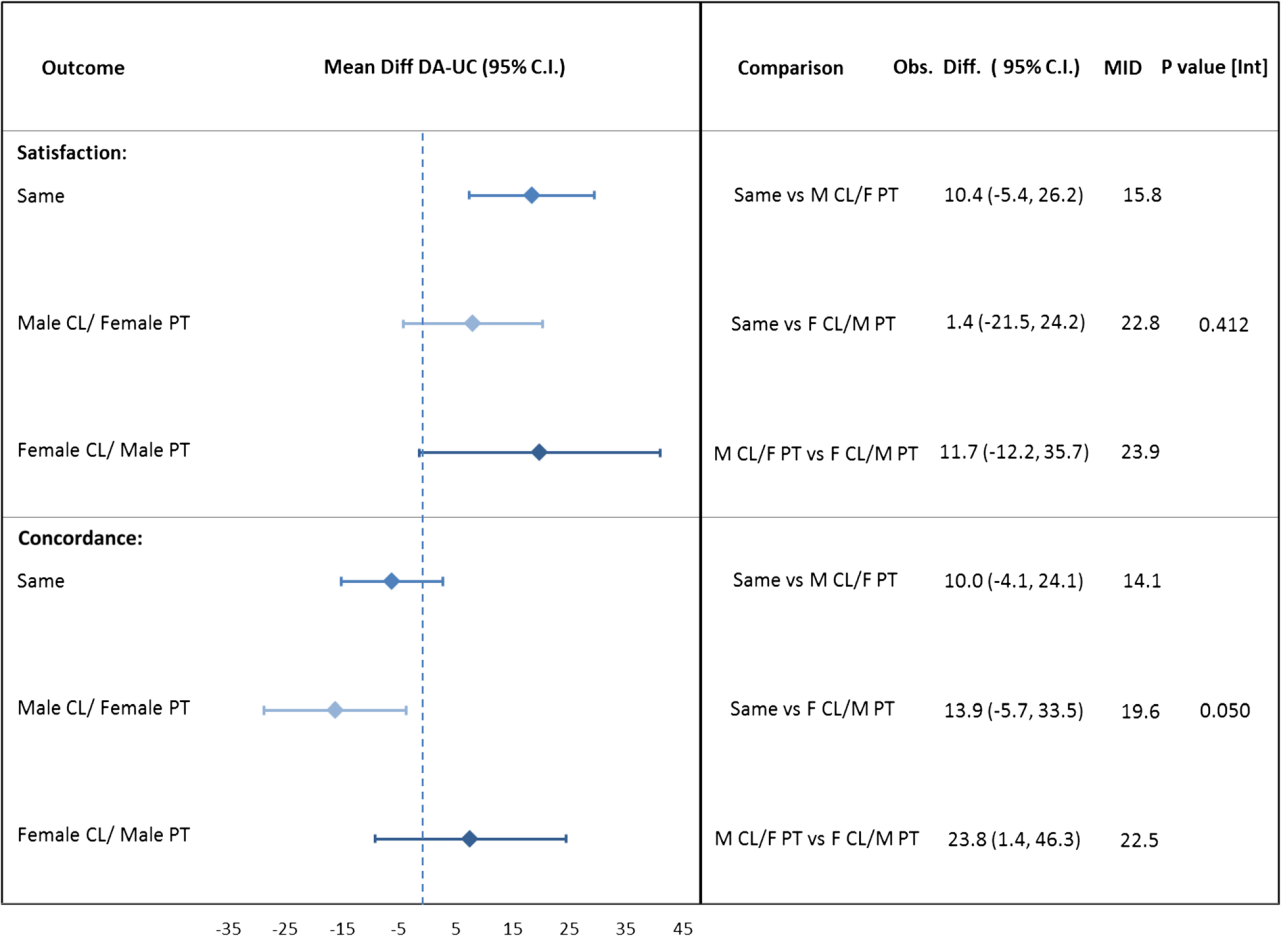
**Effect of gender groupings on patient satisfaction and concordance (agreement with decision).** Analysis separating the “same” gender group into male/male and female/female (not shown) made no change in the results. Footnote/legend: Abbreviations: Clinician CL; Patient PT; minimal important difference, MID. Mean differences (DA – UC) calculated using simulation estimates from multilevel mixed-effects logistic regression models for each arm within each gender mix. Mean differences compared between gender mixes to test against MID.

Patients were more likely to report satisfaction with the encounter in the DA arm than UC (Figure 3); however, the effect of the DA was consistent across all gender mixes. The MID for satisfaction was not met.

### Concordance

On average, a lower percentage of patients were concordant with the decision they made (i.e., they carried out the decision stated in the encounter) within the DA arm than UC (Figure 3). Among encounters in which the gender mix was same gender, there was on average 6% fewer encounters with concordance whereas the male clinician/female patient encounters had on average 16% fewer encounters with concordance. The encounters with female clinician/male patient saw an increase of 8% of encounters with concordance on average in the DA arm over UC. The impact of gender mix on concordance was found to be borderline significant (p = 0.05). When comparing male clinician/female patient and female clinician/male patient, female clinician/male patient encounters were concordant in 24% (95% CI [1, 46]) more encounters, and this difference was significant.

The unadjusted rate of concordant encounters within the DA arm is 67% versus 76% in UC; this pattern was seen in all studies except AMI Choice and DAD. The unadjusted rates for the gender mix categories showed similar concordance (range 68 to 74%).

## Discussion

We were unable to detect a difference in SDM outcomes between clinician/patient gender dyads, with the exception of concordance between the stated decision and actual action taken where female clinician/male patient dyads, which showed borderline significant increased concordance with DA use and all other dyads showed a decrease (p = 0.05). These results suggest that these DAs largely work with the same efficacy across gender dyads, and purported barriers to the use of DAs at the point of care based on gender were not detected. However, this finding needs to be confirmed because the study was underpowered to detect the observed differences, as evidenced by the observed differences being less than the MID.

Putting these results in context is challenging, given a general lack of studies on gender and SDM. However, a limited number of qualitative studies on clinician and patient gender exist from which we may draw tentative comparisons.

In general, the male communication style has been characterized as task-oriented, forceful, dominant and competitive with frequent interruptions, whereas the female communication style has been generalized as emotional, subjective, polite and self-revealing with more concern and awareness of the feelings of others shown [4]. This would suggest that the female communication style might be better suited for facilitating SDM.

Female physicians have been observed to engage in more psychosocial counseling (including discussion of social and family issues), in addition to providing more subjective and objective information, acknowledgement, partnership building and question asking. Female physicians also better explore both the disease and the illness experience and involve patients more in decision making [2,20]. By contrast, male clinicians are more assertive, give more advice and interpretation, and focus more on technical aspects of the clinical consultation, such as the physical examination [2]. These observations also suggest that females may be better facilitators of SDM.

When considering patients separately, female patients also tend to ask more questions, get more information, receive more counseling, engage in more emotional statements and be more involved in clinical encounters than male patients, although these findings have not been uniform across studies [2,5]. Female patients ask more questions, present more symptoms and give more detailed medical histories compared to male patients [21]. As a result, female patients also have more patient-centered interactions with clinicians than males, suggesting that they may more readily participate in SDM [22].

A qualitative systematic review considered the four different clinician-patient dyads separately. In general, male/male dyads were considered to involve equality between the clinician and patient as well as ease of conversation when compared to gender discordant dyads [3]. It has also been observed that the content of the conversation in male/male dyads is more likely to focus on the male patient’s social agenda when compared to other dyads, and a biopsychosocial approach appears to occur more frequently in male/male dyads compared to female/female dyads [3]. Male doctor/female patient dyads seem to be least patient centered, with doctors making more assumptions in lieu of clarifying with patients and discussing self-management less [3]. Female doctor/female patient dyads include more psychosocial and biomedical language in the context of the greatest patient-centeredness of all dyads [3]. Finally, female doctor/male patient dyads appear to be tense, with unfriendly voice tones from clinicians and dominant voice tones from both parties [3]. It appears, however, that males tend to use interactions with female clinicians to address emotional concerns more than they do with male clinicians, and males who see female clinicians have more participatory visits than males who see male clinicians [2,3]. In general, it seems there is less tension around power and status within same gender dyads, compared to gender discordant dyads, and concordance is associated with greater understanding [2,3]. Based on these characteristics, it might be reasonable to hypothesize that gender concordance is important for facilitating SDM but concordance may be trumped by the effect of clinician gender; however, our study did not observe this. One potential reason that we did not observe significant effects of gender mix on shared decision making outcomes is that the qualitative differences observed in these studies do not translate to changes in SDM in the course of delivering a DA intervention to a patient, or they do but the differences are small.

### Strengths and limitations

This study has many strengths, including analyzing the impact of clinician-patient gender dyads on several SDM outcomes in DA clinical trials in a number of clinical settings (e.g., emergency department, chronic care follow-up) and contexts (i.e., academic medical center, rural clinic). However, several factors may limit the generalizability of our results. We did not perform a systematic review to identify other potentially includable studies. Our study included only clinicians in Rochester, Minnesota, USA, and the surrounding communities, and our results may not be generalizable to other settings. It is also unknown if the clinicians who participated in our clinical trials differ in their ability to overcome postulated gender dyad related barriers; that is to say that we cannot separate the effect of the DA from the effect of our clinician population on overcoming the postulated gender dyad communication barriers, and perhaps the generalized cultural stereotypes regarding dyad communication styles do not apply to the clinicians and patients who participated in our studies or do not exist at all. An alternative hypothesis is that the communication styles we believed to facilitate SDM have less of an effect than originally thought. As three of the included trials were cluster randomized trials, and the clustering effect was not taken into account, this is a limitation of our findings. The three cluster-randomized trials included (Diabetes Medication Choice, Statin Choice and DAD) either had no intra-cluster coefficient effect, or it was so negligible that no noticeable effect of the randomization would be found. Finally, while we did not detect an effect of gender dyads greater than the MID for most outcomes we examined, it is possible that a subtler signal might exist but was not detectable given the limited statistical power of our study.

For the random effects of the study parameter, the variance of the intercept for all outcomes is low and shows little change across studies except for the outcome of OPTION scores. While this may be indicative of a large variation in findings across studies, this is also the only endpoint that is adjudicated by third party reviewers and also had a lower sample size as video recordings were not available for all encounters.

### Future directions

As the SDM field begins to look past the clinician-patient dyad and we accrue more data on patients with three or more involved parties, future work will investigate the impact of gender discordance and concordance in triads and beyond. We will also increase our power as we continue to accrue data, and we anticipate our ability to detect small differences in the impact of dyad gender influences on SDM outcomes will increase over time.

## Conclusions

A difference in SDM outcomes during encounters was not detected between clinician/patient gender dyads with the exception of the outcome of concordance between stated decision and actual action taken where female clinician/male patient dyads showed borderline significant increased concordance with DA use and all other dyads showed a decrease. This suggests that gender dyads may not act as barriers to SDM during the implementation of DAs at the point of care. These results suggest but cannot confirm that these DAs used at the point of care largely work with the same efficacy across gender dyads, and purported barriers to SDM based on gender were not detected when tested for a minimum detected difference.

## Competing interests

The authors declare that they have no competing interests.

## Authors’ contributions

All authors have made substantial contributions to conception and design, acquisition, and/or analysis and interpretation of the data; have been involved in drafting or critically revising the manuscript; and have given final approval of the current version to be published. KW drafted the background and discussion, interpreted the data, and critically revised the remainder of the manuscript. MB and JE conducted the analysis and drafted the methods and results of the manuscript and critically revised the remainder of the manuscript. AL and VM substantially contributed to the design of the study and have critically revised the manuscript. EH and HT contributed to the design of included studies and the interpretation of the data and have critically revised the manuscript.

## Pre-publication history

The pre-publication history for this paper can be accessed here:

http://www.biomedcentral.com/1472-6947/14/81/prepub

## Supplementary Material

Additional file 1 Reporting checklist. Click here for file

Additional file 2 Model coefficients. Click here for file

## References

[B1] Charles C Gafni A Whelan T Shared decision-making in the medical encounter: what does it mean? (or it takes at least two to tango) Soc Sci Med 1997 44 5 681 692 10.1016/S0277-9536(96)00221-3 9032835

[B2] Bertakis KD The influence of gender on the doctor-patient interaction Patient Educ Couns 2009 76 3 356 360 10.1016/j.pec.2009.07.022 19647968

[B3] Sandhu H Adams A Singleton L Clark-Carter D Kidd J The impact of gender dyads on doctor-patient communication: a systematic review Patient Educ Couns 2009 76 3 348 355 10.1016/j.pec.2009.07.010 19647969

[B4] Aries E Canary D, Dindia K Gender Differences in Interaction: A re-Examination Sex Differences and Similarities in Communication 1998 New Jersey: Lawrence Erbaum Associates 65 81

[B5] Hall JA Roter DL Patient gender and communication with physicians: results of a community-based study Womens Health 1995 1 1 77 95 9373374

[B6] Moher D Liberati A Tetzlaff J Altman DG Preferred reporting items for systematic reviews and meta-analyses: the PRISMA statement BMJ 2009 339 b2535 10.1136/bmj.b2535 19622551 PMC2714657

[B7] Riley RD Lambert PC Abo-Zaid G Meta-analysis of individual participant data: rationale, conduct, and reporting BMJ 2010 340 c221 10.1136/bmj.c221 20139215

[B8] Hess EP Knoedler MA Shah ND Kline JA Breslin M Branda ME Pencille LJ Asplin BR Nestler DM Sadosty AT Stiell IG Ting HH Montori VM The chest pain choice decision aid: a randomized trial Circ Cardiovasc Qual Outcomes 2012 5 3 251 259 10.1161/CIRCOUTCOMES.111.964791 22496116

[B9] Branda M LeBlanc A Shah N Tiedje K Ruud K Van Houten H Pencille L Kurland M Yawn B Montori V Shared decision making for patients with type 2 diabetes: a randomized trial in primary care BMC Health Serv Res 2013 13 1 301 10.1186/1472-6963-13-301 23927490 PMC3751736

[B10] Mullan RJ Montori VM Shah ND Christianson TJ Bryant SC Guyatt GH Perestelo-Perez LI Stroebel RJ Yawn BP Yapuncich V Breslin MA Pencille L Smith SA The diabetes mellitus medication choice decision aid: a randomized trial Arch Intern Med 2009 169 17 1560 1568 19786674 10.1001/archinternmed.2009.293

[B11] Montori VM Shah ND Pencille LJ Branda ME Van Houten HK Swiglo BA Kesman RL Tulledge-Scheitel SM Jaeger TM Johnson RE Bartel GA Melton LJ 3rd Wermers RA Use of a decision aid to improve treatment decisions in osteoporosis: the osteoporosis choice randomized trial Am J Med 2011 124 6 549 556 10.1016/j.amjmed.2011.01.013 21605732

[B12] Mann DM Ponieman D Montori VM Arciniega J McGinn T The statin choice decision aid in primary care: a randomized trial Patient Educ Couns 2010 80 1 138 140 10.1016/j.pec.2009.10.008 19959322

[B13] Nannenga MR Montori VM Weymiller AJ Smith SA Christianson TJ Bryant SC Gafni A Charles C Mullan RJ Jones LA Bolona ER Guyatt GH A treatment decision aid may increase patient trust in the diabetes specialist. The statin choice randomized trial Health Expect 2009 12 1 38 44 10.1111/j.1369-7625.2008.00521.x 19250151 PMC5060475

[B14] O’Connor AM Validation of a decisional conflict scale Med Decis Making 1995 15 1 25 30 10.1177/0272989X9501500105 7898294

[B15] Elwyn G Edwards A Wensing M Hood K Atwell C Grol R Shared decision making: developing the OPTION scale for measuring patient involvement Qual Saf Health Care 2003 12 2 93 99 10.1136/qhc.12.2.93 12679504 PMC1743691

[B16] Stewart GB Altman DG Askie LM Duley L Simmonds MC Stewart LA Statistical analysis of individual participant data meta-analyses: a comparison of methods and recommendations for practice PLoS One 2012 7 10 e46042 10.1371/journal.pone.0046042 23056232 PMC3463584

[B17] Higgins JP Thompson SG Deeks JJ Altman DG Measuring inconsistency in meta-analyses BMJ 2003 327 7414 557 560 10.1136/bmj.327.7414.557 12958120 PMC192859

[B18] Norman GR Sloan JA Wyrwich KW Interpretation of changes in health-related quality of life: the remarkable universality of half a standard deviation Med Care 2003 41 5 582 592 12719681 10.1097/01.MLR.0000062554.74615.4C

[B19] Harris PA Taylor R Thielke R Payne J Gonzalez N Conde JG Research electronic data capture (REDCap)–a metadata-driven methodology and workflow process for providing translational research informatics support J Biomed Inform 2009 42 2 377 381 10.1016/j.jbi.2008.08.010 18929686 PMC2700030

[B20] Elstad JI Women’s priorities regarding physician behavior and their preference for a female physician Women Health 1994 21 4 1 19 10.1300/J013v21n04_01 7941608

[B21] Elderkin-Thompson V Waitzkin H Differences in clinical communication by gender J Gen Intern Med 1999 14 2 112 121 10.1046/j.1525-1497.1999.00296.x 10051782

[B22] Bertakis KD Franks P Epstein RM Patient-centered communication in primary care: physician and patient gender and gender concordance J Womens Health (Larchmt) 2009 18 4 539 545 10.1089/jwh.2008.0969 19361322

