# Supplementary material for: Genders of patients and clinicians and their effect on shared decision making: a participant-level meta-analysis
Source: BMC Med Inform Decis Mak. 2014 Sep 2;14:81. doi: 10.1186/1472-6947-14-81 (PMC4170214; doi:10.1186/1472-6947-14-81)
Supplement: Additional file 2 — Model coefficients. [file 1472-6947-14-81-S2.docx]

Model Coefficients

| **Outcome:** | **Satisfaction** | | | **Concordance** | | | **OPTION Score** | | |
| --- | --- | --- | --- | --- | --- | --- | --- | --- | --- |
| **Variable** | Coefficient | S.E. | p value | Coefficient | S.E. | p value | Coefficient | S.E. | p value |
| **Arm:** UC |  |  |  |  |  |  |  |  |  |
| DA | 0.864 | 0.258 | 0.001 | -0.419 | 0.281 | 0.136 | 19.368 | 1.746 | 0.000 |
|  |  |  |  |  |  |  |  |  |  |
| **Gender Mix:** Same |  |  |  |  |  |  |  |  |  |
| Male Clinician:Female Patient | 0.266 | 0.254 | 0.294 | 0.269 | 0.328 | 0.412 | -2.270 | 1.797 | 0.207 |
| Female Clinician:Male Patient | -0.066 | 0.416 | 0.874 | -0.470 | 0.461 | 0.307 | -1.867 | 3.046 | 0.540 |
|  |  |  |  |  |  |  |  |  |  |
| **Arm*Gender Mix:** UC & Any Gender Mix or DA & Same |  |  |  |  |  |  |  |  |  |
| DA & Male Clinician:Female Patient | -0.484 | 0.388 | 0.212 | -0.639 | 0.439 | 0.145 | -0.371 | 2.549 | 0.884 |
| DA & Female Clinician:Male Patient | 0.062 | 0.549 | 0.910 | 0.898 | 0.589 | 0.128 | -0.296 | 3.994 | 0.941 |
|  |  |  |  |  |  |  |  |  |  |
| **Age** | 0.012 | 0.008 | 0.148 | 0.004 | 0.009 | 0.675 | -0.067 | 0.055 | 0.225 |
|  |  |  |  |  |  |  |  |  |  |
| **Education:** HS or less |  |  |  |  |  |  |  |  |  |
| Some college/Voc. School | -0.194 | 0.214 | 0.365 | -0.014 | 0.232 | 0.953 | 2.380 | 1.459 | 0.103 |
| 4 year/Graduate Degree | -0.304 | 0.220 | 0.168 | 0.177 | 0.244 | 0.469 | 1.795 | 1.503 | 0.232 |
|  |  |  |  |  |  |  |  |  |  |
| **Clinician Type:** Staff physician |  |  |  |  |  |  |  |  |  |
| Physician in training | -0.574 | 0.224 | 0.010 | -0.140 | 0.248 | 0.573 | -1.224 | 1.401 | 0.383 |
| Nurse/NP/PA | -0.252 | 0.311 | 0.417 | 0.092 | 0.339 | 0.785 | -1.862 | 2.671 | 0.486 |
|  |  |  |  |  |  |  |  |  |  |
| **Intercept** | 0.111 | 0.569 | 0.845 | 0.821 | 0.666 | 0.218 | 26.459 | 6.971 | <0.0001 |
|  |  |  |  |  |  |  |  |  |  |
| **Random-Effects Parameters:Study** |  |  |  |  |  |  |  |  |  |
| Variance (Intercept) | 0.095 | 0.0989 |  | 0.242 | 0.171 |  | 201.207 | 118.026 |  |
| Variance (Residual) |  |  |  |  |  |  | 168.494 | 10.809 |  |

| **Outcome:** | **Knowledge** | | | **DCS Support** | | | **DCS Informed** | | |
| --- | --- | --- | --- | --- | --- | --- | --- | --- | --- |
| **Variable** | Coefficient | S.E. | p value | Coefficient | S.E. | p value | Coefficient | S.E. | p value |
| **Arm:** UC |  |  |  |  |  |  |  |  |  |
| DA | 0.132 | 0.028 | 0.000 | 3.848 | 2.123 | 0.070 | 7.740 | 2.182 | 0.000 |
|  |  |  |  |  |  |  |  |  |  |
| **Gender Mix:** Same |  |  |  |  |  |  |  |  |  |
| Male Clinician:Female Patient | 0.000 | 0.029 | 0.990 | -2.569 | 2.243 | 0.252 | -0.862 | 2.278 | 0.705 |
| Female Clinician:Male Patient | -0.027 | 0.052 | 0.607 | -4.728 | 3.860 | 0.221 | -3.164 | 3.947 | 0.423 |
|  |  |  |  |  |  |  |  |  |  |
| **Arm*Gender Mix:** UC & Any Gender Mix or DA & Same |  |  |  |  |  |  |  |  |  |
| DA & Male Clinician:Female Patient | 0.022 | 0.041 | 0.580 | 4.484 | 3.219 | 0.164 | 4.964 | 3.289 | 0.131 |
| DA & Female Clinician:Male Patient | 0.078 | 0.067 | 0.243 | 4.388 | 4.795 | 0.360 | 1.752 | 4.918 | 0.722 |
|  |  |  |  |  |  |  |  |  |  |
| **Age** | -0.001 | 0.001 | 0.264 | 0.087 | 0.065 | 0.176 | -0.020 | 0.070 | 0.775 |
|  |  |  |  |  |  |  |  |  |  |
| **Education:** HS or less |  |  |  |  |  |  |  |  |  |
| Some college/Voc. School | 0.036 | 0.024 | 0.126 | 1.357 | 1.782 | 0.446 | 1.859 | 1.810 | 0.304 |
| 4 year/Graduate Degree | 0.055 | 0.024 | 0.024 | 2.108 | 1.854 | 0.255 | -0.242 | 1.890 | 0.898 |
|  |  |  |  |  |  |  |  |  |  |
| **Clinician Type:** Staff physician |  |  |  |  |  |  |  |  |  |
| Physician in training | -0.015 | 0.024 | 0.539 | -2.097 | 1.769 | 0.236 | -2.737 | 1.960 | 0.163 |
| Nurse/NP/PA | 0.007 | 0.036 | 0.846 | 1.033 | 2.433 | 0.671 | 1.684 | 2.557 | 0.510 |
|  |  |  |  |  |  |  |  |  |  |
| **Intercept** | 0.463 | 0.067 | <0.0001 | 76.622 | 4.44 | <0.0001 | 76.816 | 4.869 | <0.0001 |
|  |  |  |  |  |  |  |  |  |  |
| **Random-Effects Parameters:Study** |  |  |  |  |  |  |  |  |  |
| Variance (Intercept) | 0.003 | 0.002 |  | 3.23*10^-14^ | 5.61*10^-13^ |  | 7.149 | 5.966 |  |
| Variance (Residual) | 0.054 | 0.003 |  | 378.655 | 20.154 |  | 390.022 | 20.799 |  |

| **Outcome:** | **DCS Effective** | | | **DCS Certain** | | | **DCS Values** | | |
| --- | --- | --- | --- | --- | --- | --- | --- | --- | --- |
| **Variable** | Coefficient | S.E. | p value | Coefficient | S.E. | p value | Coefficient | S.E. | p value |
| **Arm:** UC |  |  |  |  |  |  |  |  |  |
| DA | 1.060 | 0.398 | 0.008 | 6.982 | 2.406 | 0.004 | 10.029 | 2.469 | 0 |
|  |  |  |  |  |  |  |  |  |  |
| **Gender Mix:** Same |  |  |  |  |  |  |  |  |  |
| Male Clinician:Female Patient | -0.057 | 0.413 | 0.890 | 1.368 | 2.467 | 0.579 | 1.078 | 2.517 | 0.668 |
| Female Clinician:Male Patient | -0.471 | 0.718 | 0.511 | -6.390 | 4.405 | 0.147 | -2.937 | 4.551 | 0.519 |
|  |  |  |  |  |  |  |  |  |  |
| **Arm*Gender Mix:** UC & Any Gender Mix or DA & Same |  |  |  |  |  |  |  |  |  |
| DA & Male Clinician:Female Patient | -0.055 | 0.598 | 0.926 | -1.459 | 3.459 | 0.673 | -0.177 | 3.552 | 0.96 |
| DA & Female Clinician:Male Patient | 0.694 | 0.894 | 0.438 | 7.021 | 5.765 | 0.223 | 4.130 | 5.895 | 0.484 |
|  |  |  |  |  |  |  |  |  |  |
| **Age** | 0.029 | 0.013 | 0.022 | 0.093 | 0.070 | 0.186 | 0.029 | 0.078 | 0.711 |
|  |  |  |  |  |  |  |  |  |  |
| **Education:** HS or less |  |  |  |  |  |  |  |  |  |
| Some college/Voc. School | 0.229 | 0.329 | 0.486 | 2.109 | 2.032 | 0.299 | 1.143 | 2.081 | 0.583 |
| 4 year/Graduate Degree | 0.447 | 0.344 | 0.193 | 2.484 | 2.069 | 0.230 | -0.513 | 2.109 | 0.808 |
|  |  |  |  |  |  |  |  |  |  |
| **Clinician Type:** Staff physician |  |  |  |  |  |  |  |  |  |
| Physician in training | -0.536 | 0.360 | 0.137 | -3.430 | 1.831 | 0.061 | -2.275 | 2.025 | 0.261 |
| Nurse/NP/PA | -0.730 | 0.466 | 0.117 | -0.140 | 3.090 | 0.964 | 4.181 | 3.182 | 0.189 |
|  |  |  |  |  |  |  |  |  |  |
| **Intercept** | 94.433 | 0.903 | <0.0001 | 70.675 | 4.962 | <0.0001 | 72.997 | 5.673 | <0.0001 |
|  |  |  |  |  |  |  |  |  |  |
| **Random-Effects Parameters:Study** |  |  |  |  |  |  |  |  |  |
| Variance (Intercept) | 0.376 | 0.291 |  | 2.96*10^-12^ | 6.28*10^-11^ |  | 10.985 | 9.502 |  |
| Variance (Residual) | 12.853 | 0.686 |  | 361.076 | 21.974 |  | 374.321 | 22.889 |  |
